# Analysis of prefrontal cerebral blood volume and flow changes in ESKD patients undergoing hemodialysis using functional near-infrared spectroscopy

**DOI:** 10.1080/0886022X.2024.2387426

**Published:** 2024-08-13

**Authors:** Chang Min Heo, Jiyae Yi, Kang Min Park, Dong Ah Lee, Yoo Jin Lee, Bong Soo Park, Yang Wook Kim, Junghae Ko, Hyunwoo Kim, Sihyung Park

**Affiliations:** a Department of Internal Medicine, Inje University College of Medicine, Busan, South Korea; b Neurology, Haeundae Paik Hospital, Inje University College of Medicine, Busan, South Korea; c Department of Internal Medicine, Jeju National University Hospital, Jeju National University College of Medicine, Jeju, South Korea

**Keywords:** Hemodialysis, brain, cerebral blood volume, cerebral blood flow, near-infrared spectroscopy

## Abstract

**Background:**

End-stage kidney disease (ESKD) patients undergoing hemodialysis experience diverse neurological complications. This study investigated prefrontal cerebral blood volume (CBV) and cerebral blood flow (CBF) during hemodialysis using functional near-infrared spectroscopy (fNIRS) to analyze cerebral hemodynamic changes.

**Methods:**

ESKD patients undergoing maintenance hemodialysis without a history of neurological disorders were enrolled prospectively. The fNIRS data were collected using a NIRSIT Lite device. The fNIRS values were recorded three times for each patient: before the start of hemodialysis (pre-HD), 1 h after the start of hemodialysis (mid-HD), and after the end of hemodialysis (post-HD). The average changes in oxy-hemoglobin (HbO_2_), deoxy-hemoglobin (HbR), total hemoglobin (HbT, calculated as HbO_2_ + HbR) concentrations, and in hemoglobin concentration difference (HbD, calculated as HbO_2_ − HbR) were analyzed. We then compared the differences in changes in HbO_2_, HbR, HbT, and HbD according to the hemodialysis period.

**Results:**

Thirty hemodialysis patients were analyzed. The change in HbO_2_, HbT, and HbD levels showed significant differences according to the hemodialysis period. Between the pre-HD and post-HD periods, there were significant differences in changes in HbO_2_ (0.005 ± 0.001 µM vs. 0.015 ± 0.004 µM, *p* = .046) and HbT (0.006 ± 0.001 µM vs. 0.016 ± 0.008 µM, *p* = .029). Additionally, between pre-HD and post-HD periods, HbD tended to increase (0.005 ± 0.001 µM vs. 0.014 ± 0.004 µM, *p* = .094).

**Conclusions:**

We demonstrated that during one hemodialysis session, the relative change in prefrontal CBV increased post-HD compared with pre-HD. These results are expected to help understanding the mechanisms underlying the effects of hemodialysis on brain function.

## 1. Introduction

End-stage kidney disease (ESKD) is defined by a glomerular filtration rate <15 mL/min/1.73 m^2^, which is the most severe form of chronic kidney disease (CKD), requiring dialysis. Patients with ESKD exhibit a higher incidence of various complications, including neurological problems, compared with healthy individuals [1]. In addition, hemodialysis treatment to support kidney impairment has been reported to increase neurological complications, such as cognitive decline and cerebral infarction; moreover, white matter integrity declines with prolonged hemodialysis treatment [2–4]. Additionally, hemodialysis can also lead to cardiac injury and myocardial stunning due to hypotension and tissue ischemia because of extracorporeal circulation [5,6]. Recurrent stress has been suggested to be a mechanism that causes ischemic brain injury and neurological complications [7,8]. However, neurological complications such as dementia and cognitive disorders are more often observed in patients under-dialyzed patients [9,10].

A few studies have demonstrated the relationship between hemodialysis and blood flow in the brain. Studies estimating cerebral blood flow (CBF) using transcranial Doppler ultrasound have been inconclusive [11–13]. A recent study using positron emission tomography-computed tomography (PET-CT) revealed that CBF decreased after hemodialysis [14]. They proposed that the reduced CBF caused cerebral ischemic injury. However, studies on changes in CBF during hemodialysis are inconclusive, and the relationship remains unclear.

Functional near-infrared spectroscopy (fNIRS) can be used to evaluate brain activity by precisely measuring changes in the concentrations of oxy-hemoglobin (HbO_2_) and deoxy-hemoglobin (HbR). The fNIRS signals have been used as indicators to evaluate cerebral hemodynamics and functional connectivity. Previous studies have shown that fNIRS is a useful method for measuring relative changes in cerebral blood volume (CBV) and CBF [15–18]. The waveforms corresponding to changes in the HbO_2_ and HbR are generated by the change in light absorption according to the change in local CBV and CBF. Therefore, changes in HbO_2_ and HbR represent relative changes in CBV and CBF.

This study aimed to investigate the relationship between prefrontal CBV and CBF and hemodialysis using fNIRS and analyze the effect of hemodialysis on cerebral hemodynamics.

## 2. Methods

### 2.1. Participants: patients with ESKD

All participants provided informed consent before enrolling in the trial. This prospective study was conducted at a single university hospital between June 2022 and September 2022. The inclusion criteria were as follows: (1) ESKD with hemodialysis, (2) dialysis vintage of at least 6 months, and (3) no history of proven neurological disorders. The exclusion criteria include a dialysis vintage of less than 6 months or a history of neurological disorders. This study was approved by the regional Institutional Review Board (approval number: HPIRB 2022-6-004-003). This study was conducted on human subjects and was carried out in accordance with the Declaration of Helsinki.

For participants’ demographic data, we investigated age, sex, dialysis duration, body mass index, *Kt*/*V*, ultrafiltration volume, ultrafiltration rate, and blood pressure. Additionally, we verified the presence of comorbidities, including hypertension and diabetes. Laboratory data included measurements of hemoglobin, iron, ferritin, total iron binding capacity, transferrin saturation, albumin, β_2_-microglobulin, total cholesterol, triglyceride, high-density lipoprotein–cholesterol, low-density lipoprotein–cholesterol, calcium, phosphate, parathyroid hormone, and C-reactive protein.

### 2.2. fNIRS data acquisition

fNIRS data were collected using a NIRSIT Lite system (OBELAB Inc., Seoul, South Korea) [19]. NIRSIT Lite is an ultra-lightweight (200 g), portable, wireless system that can be used as a wearable device, such as a hairband, that measures brain perfusion status in real-time. fNIRS estimates the brain’s cortical hemodynamic activity by measuring changes in HbO_2_ and HbR using the different absorption rates of near-infrared light. There are five light sources and 13 detectors in the NIRSIT Lite system. It uses 15 channels to detect fNIRS signals in the prefrontal cortex [20]. The system uses near-infrared light at wavelengths of 780 and 850 nm. The signals were measured at a sampling rate of 8.138 Hz.

To establish a similar environment, measurements were taken three times in a 5-min resting state while looking at a tablet computer screen with a white cross on a black background. fNIRS data were collected three times during a single hemodialysis session. The first measurement was performed 30 min before hemodialysis initiation (pre-HD), the second measurement was performed 1 h after the start of hemodialysis (mid-HD), and the final measurement was performed 30 min after the end of hemodialysis (post-HD).

### 2.3. Data processing

The NIRSIT Lite Analysis Tool program (version 3.2.4) was used to process data. We acquired the relative changes in the HbO_2_ and HbR collected from each detector at 8.138 Hz using the program. Changes in hemoglobin concentrations represented relative changes, with each measurement’s starting point taken as the baseline. The average values of the changes in HbO_2_, HbR, total hemoglobin concentration (HbT, calculated as HbO_2_ + HbR), and hemoglobin concentration difference (HbD, calculated as HbO_2_ − HbR) were analyzed in patients in the pre-HD, mid-HD, and post-HD periods by calculating the average of those values.

Changes in HbT are related to relative changes in CBV [15,16] and HbD indicates cerebral intravascular oxygenation and have been shown to be an indicator of CBF [17,18].

### 2.4. Statistical analysis

We compared the differences in the changes in HbO_2_, HbR, HbT, and HbD according to the hemodialysis period using repeated-measures analysis of variance. Mauchly’s test was applied to test sphericity, and Greenhouse–Geisser correction was used based on the test results. For *post hoc* multiple comparisons, Bonferroni’s correction was applied for *p* values and confidence intervals in the analysis. In addition, correlation analyses between clinical factors and changes in HbT and HbD were performed using Pearson’s method. All statistical analyses were performed using IBM SPSS Statistics for Windows (version 25.0; IBM Corp., Armonk, NY). Statistical significance was set at *p* < .05.

## 3. Results

### 3.1. Patient demographics and clinical characteristics

**Figure 1.**
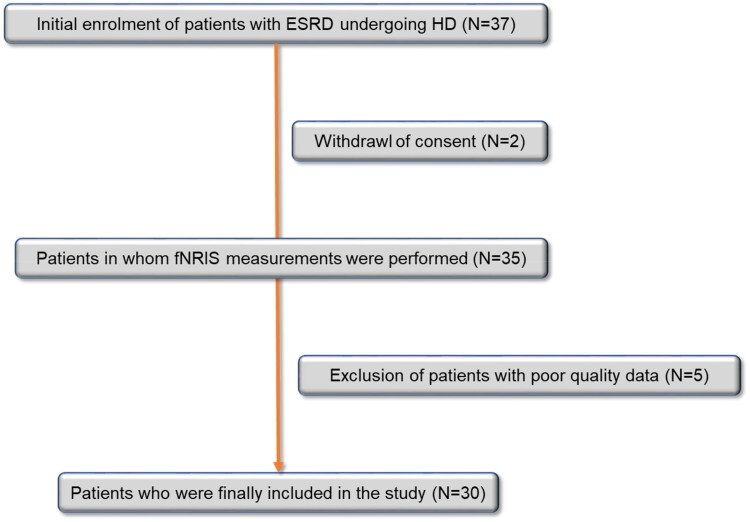
The patient selection process.

Among the 37 eligible participants, two withdrew their consent, and data from five participants were excluded because of poor quality (Figure 1). Eventually, 30 patients with ESKD who were undergoing hemodialysis were enrolled and analyzed. The mean age of the patients was 63.1 years and the mean dialysis vintage was 52.3 months. Twenty-one patients (70%) were men and 17 patients (56.7%) had diabetes. The mean hemoglobin level was 10.4 g/dL. No significant variations in blood pressure were noted during the dialysis. The demographic and clinical characteristics of the patients, including laboratory data, are presented in Table 1.

**Table 1.** Demographic and clinical characteristics of the patients.

Variables	Patients (*N* = 30)
Demographic data	
Age (years)	63.1 ± 12.8
Sex (male)	21 (70.0)
Dialysis duration, months	52.3 ± 35.0
Body mass index	23.1 ± 4.5
*Kt*/*V*	1.5 ± 0.2
Ultrafiltration volume, mL	1973.3 ± 1121.2
Ultrafiltration rate, mL/h/kg	7.9 ± 4.2
Pre-HD systolic blood pressure, mmHg	148.6 ± 14.2
Pre-HD diastolic blood pressure, mmHg	66.4 ± 12.6
Mid-HD systolic blood pressure, mmHg	144.6 ± 18.0
Mid-HD diastolic blood pressure, mmHg	64.6 ± 12.8
Post-HD systolic blood pressure, mmHg	148.6 ± 17.0
Post-HD diastolic blood pressure, mmHg	68.5 ± 16.6
Comorbidities	
Hypertension	28 (93.3)
Diabetes mellitus	17 (56.7)
Laboratory data	
Hemoglobin, g/dL	10.4 ± 1.1
Iron, μg/dL	61.8 ± 28.0
Ferritin, ng/mL	300.2 ± 260.9
Total iron binding capacity, μg/dL	239.2 ± 33.1
Transferrin saturation, %	26.5 ± 11.7
Albumin, g/dL	4.0 ± 0.3
β_2_-microglobulin, mg/dL	23.1 ± 7.0
Total cholesterol, mg/dL	129.7 ± 38.0
Triglyceride, mg/dL	109.3 ± 56.7
High-density lipoprotein–cholesterol, mg/dL	52.3 ± 20.5
Low-density lipoprotein–cholesterol, mg/dL	55.5 ± 30.0
Calcium, mg/dL	8.5 ± 0.5
Phosphate, mg/dL	4.9 ± 1.8
Parathyroid hormone, pg/mL	265.2 ± 163.6
C–reactive protein, mg/dL	0.4 ± 1.3

*Kt*/*V*: dialyzer clearance time/distribution volume of urea; HD: hemodialysis.

Data are presented as number (%) or mean ± standard deviation.

### 3.2. Differences in the changes of HbO_2_, HbR, HbD, and HbT with hemodialysis

**Figure 2.**
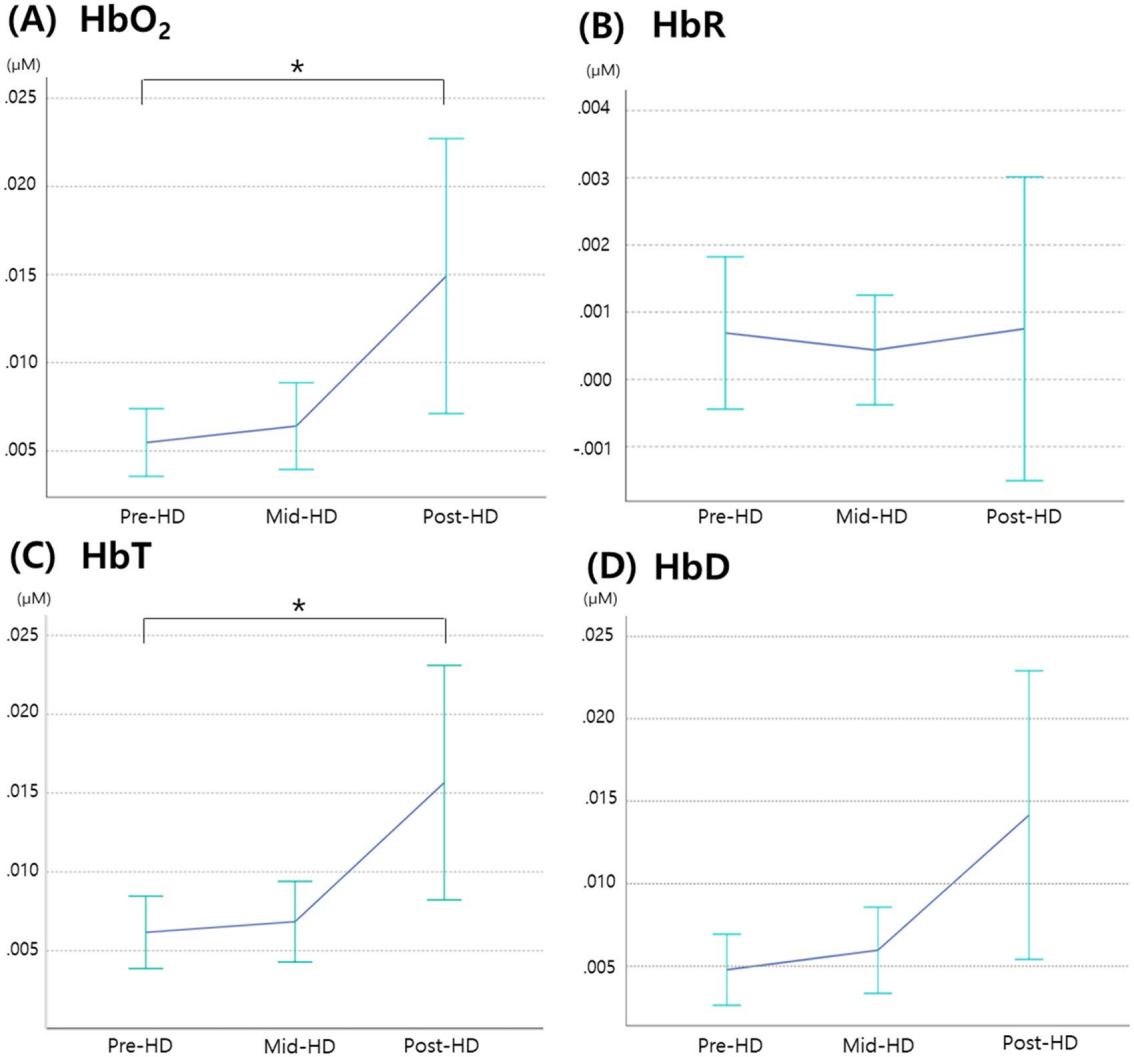
Changes in hemoglobin concentrations according to hemodialysis periods. Between the pre- and post-HD periods, there were significant differences in changes in HbO_2_ (A) and HbT (C). However, there were no statistically significant differences in the changes in HbR and HbD according to the hemodialysis period (B, D). HbO_2_: oxy-hemoglobin concentration; HbR: deoxy-hemoglobin concentration; HbT: total hemoglobin concentration; HbD: hemoglobin concentration difference; HD: hemodialysis. *Statistical significance (*p* < .05).

Table 2 and Figure 2 show changes in HbO_2_, HbR, HbT, and HbD according to hemodialysis periods. The change in HbO_2_ levels showed significant differences across time points (*F* = 5.658, *p* = .019). Between the pre-HD and post-HD periods, there were significant differences in changes in HbO_2_ (0.005 ± 0.001 µM vs. 0.015 ± 0.004 µM, *p* = .046). The change in HbT levels showed significant differences across time points (*F* = 6.373, *p* = .011). Between the pre-HD and post-HD periods, there were significant differences in changes in HbT (0.006 ± 0.001 µM vs. 0.016 ± 0.008 µM, *p* = .029). The change in HbD levels showed significant differences across time points (*F* = 4.258, *p* = .041). Between pre-HD and post-HD periods, the change in HbD levels increased; however, this was not statistically significant (0.005 ± 0.001 µM vs. 0.014 ± 0.004 µM, *p* = .094). There were no statistically significant differences between the changes in HbR concentrations according to the hemodialysis period.

**Table 2.** Changes in fNIRS data according to hemodialysis periods and differences.

Measures	Acquisition time	Changes in concentrations (µM)	*F*	*p* Value	Pairwise comparisons	*p* Value	95% confidence interval
HbO_2_	Pre-HD	0.005 ± 0.001	5.658	.019*	Mid-HD	1.000	–0.004 to 0.002
					Post-HD	.046*	–0.018 to 0.000
	Mid-HD	0.006 ± 0.001			Post-HD	.089	–0.018 to 0.001
	Post-HD	0.015 ± 0.004					
HbR	Pre-HD	0.001 ± 0.001	0.058	.912	Mid-HD	1.000	–0.002 to 0.002
					Post-HD	1.000	–0.003 to 0.003
	Mid-HD	0.000 ± 0.000			Post-HD	1.000	–0.003 to 0.003
	Post-HD	0.001 ± 0.001					
HbT	Pre-HD	0.006 ± 0.001	6.373	.011*	Mid-HD	1.000	–0.004 to 0.003
					Post-HD	.029*	–0.018 to −0.001
	Mid-HD	0.007 ± 0.001			Post-HD	.058	–0.018 to 0.000
	Post-HD	0.016 ± 0.008					
HbD	Pre-HD	0.005 ± 0.001	4.258	.041*	Mid-HD	1.000	–0.005 to 0.002
					Post-HD	.094	−0.020 to 0.001
	Mid-HD	0.006 ± 0.001			Post-HD	.183	−0.019 to 0.002
	Post-HD	0.014 ± 0.004					

fNIRS: functional near-infrared spectroscopy; HbO_2_: oxy-hemoglobin; HbR: deoxy-hemoglobin; HbT: total hemoglobin; HbD: hemoglobin difference; HD: hemodialysis.

* Statistical significance (*p* < .05).

For information regarding changes in HbO_2_, HbR, HbD, and HbT that are linked to hemodialysis in each of the 15 channels, refer to the supplementary material (Supplementary Table 1).

### 3.3. Association between clinical factors and changes in HbO_2_, HbR, HbD, and HbT

There were no definite clinical factors related to age, sex, lipid profile, or comorbidities based on fNIRS values. The remaining parameters are listed in Supplementary Table 2.

## 4. Discussion

This study investigated the effects of hemodialysis on prefrontal cerebral hemodynamics using fNIRS. Herein, this study demonstrated significant changes in HbO_2_, HbT, and HbD levels according to the hemodialysis period. We observed significantly higher HbO_2_ and HbT post-HD than pre-HD.

When a specific cerebral region becomes active, the blood flow to that region increases, resulting in an increase in HbO_2_ and a decrease in HbR. Changes in HbT are indicative of changes in CBV if the hematocrit remains constant [15,16]. As hemoglobin oxygenation causes an increase in HbO_2_ and a decrease in HbR, cerebral hemoglobin oxygenation increases HbD. Because changes in CBF alter cerebral venous oxygen saturation according to Fick’s principle, HbD and CBF have a physiologically reasonable relationship [17]. HbD has been demonstrated as a surrogate marker of CBF in previous studies, assuming that oxygen consumption does not change [17,18,21,22]. Therefore, from these study findings, we can infer significant changes in prefrontal CBV and CBF according to the hemodialysis period. Particularly, it can be interpreted that prefrontal CBV significantly increases post-HD compared to pre-HD.

The CBV refers to the total volume of blood in the brain at a given time. In contrast, CBF represents the amount of blood flow through the brain per unit time. There is a strong positive correlation between CBV and CBF and both depend on several important variables, such as cerebrovascular resistance (CVR), intracranial pressure, and mean arterial pressure [23]. CBF is determined by cerebral perfusion pressure (CPP) and CVR [CBF = CPP/CVR]. CVR can be altered by changes that occur during dialysis, such as changes in metabolites and blood viscosity. Uremic toxins such as phosphate, p-cresyl sulfate, indoxyl sulfate, fibroblast growth factor 23, asymmetric dimethylarginine, symmetric dimethylarginine, and advanced glycation products are known to cause vascular dysfunction [24]. Hemodialysis lowers CVR by lowering uremic toxin concentrations and improving vascular dysfunction. However, hemodialysis causes blood hyper-viscosity due to the effects of ultrafiltration [25]. An increase in blood viscosity increases systemic vascular resistance [26]. The correlation between ultrafiltration rate and hemoglobin is not very clear, but there is evidence suggesting that when the rate exceeds 10 mL/h/kg, it acts as a risk factor associated with the increase in post-Hb level [27]. Herein, our average ultrafiltration rate was 7.9 mL/h/kg. Although parameters such as changes in hemoglobin concentrations, which represent blood viscosity, were not measured during hemodialysis, this amount of ultrafiltration may allow the uremic toxin removal effect of hemodialysis to have a greater impact on CVR than an increase in blood viscosity, considering that the CBV increased after hemodialysis. CPP, one of the factors determining CBF, is defined as the difference between mean arterial pressure and intracranial pressure [28]. A decrease in mean arterial pressure caused by events such as hypotension during dialysis may lower CBF. Decreased systolic and mean blood pressures after dialysis, despite similar serum hemoglobin levels, resulted in a decline in CBF in a study using PET-CT [14]. Herein, we observed increased prefrontal CBV and CBF because no patient developed hypotension during hemodialysis and no changes in blood pressure were observed between hemodialysis periods. However, it should be noted that the patients were younger than the previous group (75.4 ± 5.2 years vs. 63.1 ± 12 years). If adequate ultrafiltration and stable blood pressure are maintained during dialysis, hemodialysis can positively affect CBF.

Our study had several limitations. First, this was a single-center study, and the number of subjects was small. More meaningful results could be obtained if a large number of participants were targeted at multiple institutions. Second, the study did not measure changes in hematocrit with each hemodialysis session. In order to utilize changes in HbT as a surrogate marker for CBV, it is necessary for hematocrit to remain constant. Fluctuations in hematocrit due to hemodialysis should be considered when interpreting the results. However, since the average ultrafiltration rate was low at 7.9 mL/h/kg and refill from the interstitial space to the vascular space occurred, it is expected that the impact on hematocrit variation would be minimal. Third, HbD provides a relative measure of CBF; however, it is determined by several factors such as blood flow, blood volume, metabolic rate of oxygen, capillary density, and hematocrit [29]. Therefore, the HbD used in this study may not accurately reflect CBF dynamics owing to other factors. Methodological supplementation is required to measure CBF in future studies. Fourth, the NIRSIT Lite machine could only collect fNIRS data from the frontal lobe. This study may have limitations in comprehensively assessing hemodynamics throughout the entire brain. However, because cognitive dysfunction is primarily associated with the frontal lobe, the study results may provide insights into the effects of hemodialysis on the prefrontal area, which is associated with neurologic complications in ESKD patients [30,31]. Fifth, fNIRS is a tool that indirectly estimates CBV and CBF by measuring HbO_2_ and HbR in the microvascular region. As it is not a direct measurement method, there may be limitations. Nevertheless, fNIRS is a measurement tool with several advantages, including being noninvasive, safe, portable, and capable of repetitive measurements. With proper control of various variables, fNIRS is expected to be significantly used as a surrogate marker for CBV and CBF.

In conclusion, we showed that a single hemodialysis session increased the changes in HbO_2_, HbT, and HbD levels. Between the pre-HD and post-HD periods, there were significant differences in changes in HbO_2_ and HbT. This implies that a single hemodialysis session increases CBV and CBF over time, particularly indicating an increase in CBV between pre-HD and post-HD. Hemodynamically stabilized hemodialysis sessions can positively affect prefrontal CBF and CBV through effective uremic toxin removal. These results are expected to clarify the mechanism underlying the effect of hemodialysis on brain function. Further studies are needed to overcome the limitations of our study. This study is meaningful because it is the first to use fNIRS, which is a convenient, noninvasive, easy-to-use, and validated method for measuring prefrontal CBV and CBF in hemodialysis patients.

## Supplementary Material

Supplementary table 2.docx

supplementary table 1.docx

## Data Availability

The data presented in this study are available on request from the corresponding author.
