# Supplementary material for: Analysis of prefrontal cerebral blood volume and flow changes in ESKD patients undergoing hemodialysis using functional near-infrared spectroscopy
Source: Ren Fail. 2024 Aug 13;46(2):2387426. doi: 10.1080/0886022X.2024.2387426 (PMC11328595; doi:10.1080/0886022X.2024.2387426)
Supplement: Supplementary table 2.docx [file IRNF_A_2387426_SM4078.docx]

Supplementary table 2. Correlation analysis of the changes in hemoglobin concentration and clinical factors

| Measures |  | Correlation coefficient (r) & p-value | | |
| --- | --- | --- | --- | --- |
|  | Changes in HbO_2_ | Changes in HbR | Changes in HbT | Changes in HbD |
| Dialysis duration | r=-0.168, p=0.376 | r=0.127, p=0.505 | r=-0.137, p=0.469 | r=-0.182, p=0.335 |
| Kt/V | r=-0.322, p=0.082 | r=0.268, p=0.152 | r=-0.257, p=0.171 | r=-0.357, p=0.053 |
| Ultrafiltration volume | r=0.310, p=0.096 | r=0.028, p=0.882 | r=0.333, p=0.072 | r=0.269, p=0.151 |
| Pre-HD SBP | r=0.033, p=0.861 | r=-0.108, p=0.569 | r=0.002, p=0.991 | r=0.058, p=0.762 |
| Pre-HD DBP | r=-0.126, p=0.507 | r=-0.176, p=0.352 | r=-0.186, p=0.326 | r=-0.067, p=0.726 |
| Mid-HD SBP | r=-0.141, p=0.458 | r=-0.053, p=0.779 | r=-0.164, p=0.387 | r=-0.112, p=0.557 |
| Mid-HD DBP | r=-0.009, p=0.962 | r=-0.049, p=0.795 | r=-0.024, p=0.898 | r=0.005, p=0.980 |
| Post-HD SBP | r=-0.049, p=0.798 | r=-0.223, p=0.236 | r=-0.119, p=0.532 | r=0.014, p=0.941 |
| Post-HD DBP | r=-0.054, p=0.777 | r=-0.236, p=0.209 | r=-0.128, p=0.499 | r=0.013, p=0.946 |
| Hemoglobin | r=-0.074, p=0.696 | r=0.023, p=0.903 | r=-0.071, p=0.709 | r=-0.072, p=704 |
| Albumin | r=-0.131, p=0.492 | r=-0.101, p=0.597 | r=-0.167, p=0.376 | r=-0.090, p=0.635 |
| β2–microglobulin | r=0.112, p=0.557 | r=0.054, p=0.778 | r=0.133, p=0.482 | r=0.086, p=0.653 |
| Calcium | r=-0.283, p=0.129 | r=0.294, p=0.114 | r=-0.208, p=0.271 | r=-0.328, p=0.076 |
| Phosphate | r=0.164, p=0.388 | r=0.022, p=0.910 | r=0.178, p=0.347 | r=0.140, p=0.460 |
| Parathyroid hormone | r=0.038, p=0.846 | r=0.248, p=0.194 | r=0.107, p=0.580 | r=-0.025, p=0.899 |
| C–reactive protein | r=-0.156, p=0.412 | r=-0.009, p=0.963 | r=-0.166, p=0.381 | r=-0.136, p=0.472 |

HbO_2:_ oxyhemoglobin; HbR, deoxyhemoglobin; HbT, total hemoglobin; HbD, hemoglobin difference; Kt/V, dialyzer clearance time/distribution volume of urea; HD, hemodialysis; SBP, systolic blood pressure; DBP, diastolic blood pressure; HDL, high-density lipoproteincholesterol; LDL, low-density lipoproteincholesterol
