# Supplementary material for: Analysis of prefrontal cerebral blood volume and flow changes in ESKD patients undergoing hemodialysis using functional near-infrared spectroscopy
Source: Ren Fail. 2024 Aug 13;46(2):2387426. doi: 10.1080/0886022X.2024.2387426 (PMC11328595; doi:10.1080/0886022X.2024.2387426)
Supplement: supplementary table 1.docx [file IRNF_A_2387426_SM4076.docx]

Supplemantary table 1. Changes in fNIRs data according to hemodialysis periods and differences of the 15 channels.

1) channel 1

| Measures | Acquisition time | Changes in concentrations (µM) | F | *p-value* | Pairwise comparisons | *p-value* | 95% confidence interval |
| --- | --- | --- | --- | --- | --- | --- | --- |
| HbO_2_ | Pre–HD | 0.009±0.002 | 4.451 | *0.025 | Mid–HD | 1.000 | –0.008 to 0.008 |
|  |  |  |  |  | Post–HD | 0.080 | –0.025 to 0.001 |
|  | Mid–HD | 0.009±0.003 |  |  | Post-HD | 0.095 | –0.025 to 0.001 |
|  | Post–HD | 0.021±0.005 |  |  |  | | |
| HbR | Pre–HD | –0.004±0.001 | 1.745 | 0.184 | Mid–HD | 0.322 | –0.008 to 0.002 |
|  |  |  |  |  | Post–HD | 1.000 | –0.005 to 0.006 |
|  | Mid–HD | 0.000±0.002 |  |  | Post-HD | 0.348 | –0.002 to 0.009 |
|  | Post–HD | –0.004±0.002 |  |  |  | | |
| HbT | Pre–HD | 0.006±0.001 | 4.880 | *0.022 | Mid–HD | 0.512 | –0.010 to 0.003 |
|  |  |  |  |  | Post–HD | *0.022 | –0.022 to -0.001 |
|  | Mid–HD | 0.009±0.002 |  |  | Post-HD | 0.265 | –0.020 to 0.004 |
|  | Post–HD | 0.017±0.004 |  |  |  | | |
| HbD | Pre–HD | 0.013±0.004 | 3.634 | *0.033 | Mid–HD | 1.000 | –0.009 to 0.015 |
|  |  |  |  |  | Post–HD | 0.234 | -0.030 to 0.005 |
|  | Mid–HD | 0.010±0.004 |  |  | Post-HD | 0.077 | -0.033 to 0.001 |
|  | Post–HD | 0.025±0.006 |  |  |  | | |

fNIRs, functional near-infrared spectroscopy; HbO2, oxyhemoglobin; HbR, deoxyhemoglobin; HbT, total hemoglobin; HbD, hemoglobin difference; HD, hemodialysis.

* indicates statistical significance (p<0.05)

2) channel 2

| Measures | Acquisition time | Changes in concentrations (µM) | F | *p-value* | Pairwise comparisons | *p-value* | 95% confidence interval |
| --- | --- | --- | --- | --- | --- | --- | --- |
| HbO_2_ | Pre–HD | 0.006±0.001 | 1.202 | 0.303 | Mid–HD | 0.745 | –0.018 to 0.007 |
|  |  |  |  |  | Post–HD | 0.242 | –0.019 to 0.003 |
|  | Mid–HD | 0.011±0.005 |  |  | Post-HD | 1.000 | –0.018 to 0.014 |
|  | Post–HD | 0.013±0.004 |  |  |  | | |
| HbR | Pre–HD | 0.001±0.002 | 1.463 | 0.240 | Mid–HD | 0.616 | –0.004 to 0.011 |
|  |  |  |  |  | Post–HD | 0.329 | –0.002 to 0.011 |
|  | Mid–HD | –0.003±0.002 |  |  | Post-HD | 1.000 | –0.006 to 0.008 |
|  | Post–HD | –0.003±0.002 |  |  |  | | |
| HbT | Pre–HD | 0.007±0.003 | 0.312 | 0.733 | Mid–HD | 1.000 | –0.014 to 0.010 |
|  |  |  |  |  | Post–HD | 1.000 | –0.013 to 0.007 |
|  | Mid–HD | 0.009±0.003 |  |  | Post-HD | 1.000 | –0.012 to 0.009 |
|  | Post–HD | 0.010±0.003 |  |  |  | | |
| HbD | Pre–HD | 0.004±0.002 | 1.598 | 0.215 | Mid–HD | 0.462 | –0.026 to 0.007 |
|  |  |  |  |  | Post–HD | 0.152 | -0.027 to 0.003 |
|  | Mid–HD | 0.014±0.006 |  |  | Post-HD | 1.000 | -0.025 to 0.019 |
|  | Post–HD | 0.016±0.006 |  |  |  | | |

* indicates statistical significance (p<0.05)

3) channel 3

| Measures | Acquisition time | Changes in concentrations (µM) | F | *p-value* | Pairwise comparisons | *p-value* | 95% confidence interval |
| --- | --- | --- | --- | --- | --- | --- | --- |
| HbO_2_ | Pre–HD | 0.004±0.002 | 7.232 | *0.011 | Mid–HD | 1.000 | –0.003 to 0.002 |
|  |  |  |  |  | Post–HD | *0.030 | –0.035 to –0.001 |
|  | Mid–HD | 0.005±0.001 |  |  | Post-HD | *0.039 | –0.035 to –0.001 |
|  | Post–HD | 0.023±0.007 |  |  |  | | |
| HbR | Pre–HD | 0.001±0.002 | 4.015 | *0.024 | Mid–HD | 1.000 | –0.007 to 0.006 |
|  |  |  |  |  | Post–HD | 0.090 | –0.001 to 0.014 |
|  | Mid–HD | 0.001±0.001 |  |  | Post-HD | 0.055 | 0.000 to 0.013 |
|  | Post–HD | –0.006±0.002 |  |  |  | | |
| HbT | Pre–HD | 0.005±0.002 | 4.533 | *0.031 | Mid–HD | 1.000 | –0.007 to 0.005 |
|  |  |  |  |  | Post–HD | 0.094 | –0.025 to 0.001 |
|  | Mid–HD | 0.006±0.002 |  |  | Post-HD | 0.111 | –0.024 to 0.002 |
|  | Post–HD | 0.017±0.005 |  |  |  | | |
| HbD | Pre–HD | 0.003±0.003 | 7.338 | *0.008 | Mid–HD | 1.000 | –0.009 to 0.007 |
|  |  |  |  |  | Post–HD | *0.025 | –0.048 to –0.003 |
|  | Mid–HD | 0.004±0.002 |  |  | Post-HD | *0.030 | –0.047 to –0.002 |
|  | Post–HD | 0.028±0.009 |  |  |  | | |

* indicates statistical significance (p<0.05)

4) channel 4

| Measures | Acquisition time | Changes in concentrations (µM) | F | *p-value* | Pairwise comparisons | *p-value* | 95% confidence interval |
| --- | --- | --- | --- | --- | --- | --- | --- |
| HbO_2_ | Pre–HD | 0.004±0.001 | 1.295 | 0.276 | Mid–HD | 1.000 | –0.003 to 0.003 |
|  |  |  |  |  | Post–HD | 0.269 | –0.004 to 0.001 |
|  | Mid–HD | 0.004±0.001 |  |  | Post-HD | 0.800 | –0.006 to 0.002 |
|  | Post–HD | 0.005±0.002 |  |  |  | | |
| HbR | Pre–HD | 0.002±0.001 | 0.440 | 0.577 | Mid–HD | 0.998 | –0.002 to 0.004 |
|  |  |  |  |  | Post–HD | 1.000 | –0.003 to 0.005 |
|  | Mid–HD | 0.000±0.000 |  |  | Post-HD | 1.000 | –0.003 to 0.002 |
|  | Post–HD | 0.001±0.001 |  |  |  | | |
| HbT | Pre–HD | 0.005±0.002 | 0.896 | 0.414 | Mid–HD | 1.000 | –0.003 to 0.006 |
|  |  |  |  |  | Post–HD | 1.000 | –0.005 to 0.003 |
|  | Mid–HD | 0.004±0.001 |  |  | Post-HD | 0.596 | –0.006 to 0.002 |
|  | Post–HD | 0.006±0.001 |  |  |  | | |
| HbD | Pre–HD | 0.002±0.001 | 0.846 | 0.434 | Mid–HD | 1.000 | –0.005 to 0.003 |
|  |  |  |  |  | Post–HD | 0.791 | –0.009 to 0.003 |
|  | Mid–HD | 0.003±0.001 |  |  | Post-HD | 1.000 | –0.007 to 0.004 |
|  | Post–HD | 0.005±0.002 |  |  |  | | |

* indicates statistical significance (p<0.05)

5) channel 5

| Measures | Acquisition time | Changes in concentrations (µM) | F | *p-value* | Pairwise comparisons | *p-value* | 95% confidence interval |
| --- | --- | --- | --- | --- | --- | --- | --- |
| HbO_2_ | Pre–HD | 0.003±0.001 | 1.079 | 0.327 | Mid–HD | 1.000 | –0.003 to 0.002 |
|  |  |  |  |  | Post–HD | 0.678 | –0.006 to 0.002 |
|  | Mid–HD | 0.003±0.001 |  |  | Post-HD | 1.000 | –0.006 to 0.003 |
|  | Post–HD | 0.005±0.002 |  |  |  | | |
| HbR | Pre–HD | 0.001±0.000 | 2.467 | 0.122 | Mid–HD | 0.367 | –0.001 to 0.003 |
|  |  |  |  |  | Post–HD | 0.577 | –0.007 to 0.002 |
|  | Mid–HD | 0.000±0.000 |  |  | Post-HD | 0.274 | –0.009 to 0.002 |
|  | Post–HD | 0.003±0.002 |  |  |  | | |
| HbT | Pre–HD | 0.004±0.001 | 4.211 | *0.037 | Mid–HD | 1.000 | –0.002 to 0.003 |
|  |  |  |  |  | Post–HD | 0.160 | –0.010 to 0.001 |
|  | Mid–HD | 0.003±0.001 |  |  | Post-HD | 0.095 | –0.011 to 0.001 |
|  | Post–HD | 0.008±0.002 |  |  |  | | |
| HbD | Pre–HD | 0.002±0.001 | 0.305 | 0.628 | Mid–HD | 0.656 | –0.004 to 0.001 |
|  |  |  |  |  | Post–HD | 1.000 | –0.007 to 0.007 |
|  | Mid–HD | 0.003±0.001 |  |  | Post-HD | 1.000 | –0.006 to 0.009 |
|  | Post–HD | 0.002±0.003 |  |  |  | | |

* indicates statistical significance (p<0.05)

6) channel 6

| Measures | Acquisition time | Changes in concentrations (µM) | F | *p-value* | Pairwise comparisons | *p-value* | 95% confidence interval |
| --- | --- | --- | --- | --- | --- | --- | --- |
| HbO_2_ | Pre–HD | 0.006±0.001 | 3.721 | 0.060 | Mid–HD | 1.000 | –0.007 to 0.003 |
|  |  |  |  |  | Post–HD | 0.155 | –0.040 to 0.005 |
|  | Mid–HD | 0.008±0.002 |  |  | Post-HD | 0.217 | –0.038 to 0.006 |
|  | Post–HD | 0.024±0.009 |  |  |  | | |
| HbR | Pre–HD | 0.001±0.001 | 1.375 | 0.259 | Mid–HD | 0.723 | –0.005 to 0.002 |
|  |  |  |  |  | Post–HD | 0.495 | –0.010 to 0.003 |
|  | Mid–HD | 0.003±0.001 |  |  | Post-HD | 1.000 | –0.009 to 0.004 |
|  | Post–HD | 0.005±0.003 |  |  |  | | |
| HbT | Pre–HD | 0.007±0.002 | 3.781 | 0.056 | Mid–HD | 0.860 | –0.011 to 0.004 |
|  |  |  |  |  | Post–HD | 0.128 | –0.048 to 0.004 |
|  | Mid–HD | 0.010±0.003 |  |  | Post-HD | 0.242 | –0.044 to 0.007 |
|  | Post–HD | 0.029±0.011 |  |  |  | | |
| HbD | Pre–HD | 0.005±0.001 | 3.062 | 0.088 | Mid–HD | 1.000 | –0.004 to 0.004 |
|  |  |  |  |  | Post–HD | 0.274 | –0.035 to 0.006 |
|  | Mid–HD | 0.005±0.001 |  |  | Post-HD | 0.253 | –0.034 to 0.006 |
|  | Post–HD | 0.019±0.008 |  |  |  | | |

* indicates statistical significance (p<0.05)

7) channel 7

| Measures | Acquisition time | Changes in concentrations (µM) | F | *p-value* | Pairwise comparisons | *p-value* | 95% confidence interval |
| --- | --- | --- | --- | --- | --- | --- | --- |
| HbO_2_ | Pre–HD | 0.002±0.001 | 3.630 | 0.063 | Mid–HD | 0.576 | –0.003 to 0.001 |
|  |  |  |  |  | Post–HD | 0.150 | –0.018 to 0.002 |
|  | Mid–HD | 0.003±0.001 |  |  | Post-HD | 0.259 | –0.016 to 0.003 |
|  | Post–HD | 0.010±0.004 |  |  |  | | |
| HbR | Pre–HD | 0.001±0.001 | 0.618 | 0.468 | Mid–HD | 1.000 | –0.002 to 0.002 |
|  |  |  |  |  | Post–HD | 1.000 | –0.007 to 0.003 |
|  | Mid–HD | 0.001±0.001 |  |  | Post-HD | 1.000 | –0.007 to 0.004 |
|  | Post–HD | 0.003±0.002 |  |  |  | | |
| HbT | Pre–HD | 0.003±0.001 | 4.282 | *0.043 | Mid–HD | 0.724 | –0.005 to 0.002 |
|  |  |  |  |  | Post–HD | 0.113 | –0.021 to 0.002 |
|  | Mid–HD | 0.005±0.001 |  |  | Post-HD | 0.164 | –0.019 to 0.002 |
|  | Post–HD | 0.013±0.005 |  |  |  | | |
| HbD | Pre–HD | 0.002±0.001 | 1.643 | 0.210 | Mid–HD | 1.000 | –0.004 to 0.002 |
|  |  |  |  |  | Post–HD | 0.493 | –0.017 to 0.005 |
|  | Mid–HD | 0.002±0.001 |  |  | Post-HD | 0.765 | –0.017 to 0.006 |
|  | Post–HD | 0.008±0.005 |  |  |  | | |

* indicates statistical significance (p<0.05)

8) channel 8

| Measures | Acquisition time | Changes in concentrations (µM) | F | *p-value* | Pairwise comparisons | *p-value* | 95% confidence interval |
| --- | --- | --- | --- | --- | --- | --- | --- |
| HbO_2_ | Pre–HD | 0.005±0.002 | 1.439 | 0.244 | Mid–HD | 0.749 | –0.013 to 0.005 |
|  |  |  |  |  | Post–HD | 0.134 | –0.019 to 0.002 |
|  | Mid–HD | 0.009±0.004 |  |  | Post-HD | 1.000 | –0.021 to 0.013 |
|  | Post–HD | 0.014±0.005 |  |  |  | | |
| HbR | Pre–HD | 0.000±0.001 | 0.674 | 0.428 | Mid–HD | 1.000 | –0.001 to 0.002 |
|  |  |  |  |  | Post–HD | 1.000 | –0.010 to 0.005 |
|  | Mid–HD | –0.001±0.001 |  |  | Post-HD | 1.000 | –0.011 to 0.006 |
|  | Post–HD | 0.002±0.003 |  |  |  | | |
| HbT | Pre–HD | 0.005±0.002 | 1.614 | 0.215 | Mid–HD | 0.772 | –0.012 to 0.004 |
|  |  |  |  |  | Post–HD | 0.281 | –0.027 to 0.005 |
|  | Mid–HD | 0.009±0.004 |  |  | Post-HD | 1.000 | –0.027 to 0.013 |
|  | Post–HD | 0.016±0.007 |  |  |  | | |
| HbD | Pre–HD | 0.005±0.003 | 0.772 | 0.400 | Mid–HD | 0.764 | –0.015 to 0.006 |
|  |  |  |  |  | Post–HD | 0.296 | –0.015 to 0.003 |
|  | Mid–HD | 0.010±0.004 |  |  | Post-HD | 1.000 | –0.019 to 0.017 |
|  | Post–HD | 0.011±0.005 |  |  |  | | |

* indicates statistical significance (p<0.05)

9) channel 9

| Measures | Acquisition time | Changes in concentrations (µM) | F | *p-value* | Pairwise comparisons | *p-value* | 95% confidence interval |
| --- | --- | --- | --- | --- | --- | --- | --- |
| HbO_2_ | Pre–HD | 0.004±0.001 | 2.251 | 0.114 | Mid–HD | 1.000 | –0.006 to 0.003 |
|  |  |  |  |  | Post–HD | 0.168 | –0.011 to 0.001 |
|  | Mid–HD | 0.006±0.002 |  |  | Post-HD | 0.666 | –0.010 to 0.003 |
|  | Post–HD | 0.009±0.003 |  |  |  | | |
| HbR | Pre–HD | 0.001±0.001 | 0.984 | 0.347 | Mid–HD | 1.000 | –0.002 to 0.003 |
|  |  |  |  |  | Post–HD | 1.000 | –0.009 to 0.004 |
|  | Mid–HD | 0.001±0.001 |  |  | Post-HD | 0.862 | –0.010 to 0.004 |
|  | Post–HD | 0.004±0.003 |  |  |  | | |
| HbT | Pre–HD | 0.006±0.002 | 3.097 | 0.066 | Mid–HD | 1.000 | –0.007 to 0.005 |
|  |  |  |  |  | Post–HD | 0.119 | –0.016 to 0.001 |
|  | Mid–HD | 0.006±0.002 |  |  | Post-HD | 0.297 | –0.016 to 0.003 |
|  | Post–HD | 0.013±0.004 |  |  |  | | |
| HbD | Pre–HD | 0.003±0.001 | 0.282 | 0.673 | Mid–HD | 0.915 | –0.007 to 0.003 |
|  |  |  |  |  | Post–HD | 1.000 | –0.012 to 0.007 |
|  | Mid–HD | 0.005±0.002 |  |  | Post-HD | 1.000 | –0.010 to 0.010 |
|  | Post–HD | 0.005±0.004 |  |  |  | | |

* indicates statistical significance (p<0.05)

10) channel 10

| Measures | Acquisition time | Changes in concentrations (µM) | F | *p-value* | Pairwise comparisons | *p-value* | 95% confidence interval |
| --- | --- | --- | --- | --- | --- | --- | --- |
| HbO_2_ | Pre–HD | 0.005±0.002 | 1.619 | 0.207 | Mid–HD | 1.000 | –0.006 to 0.006 |
|  |  |  |  |  | Post–HD | 0.481 | –0.011 to 0.003 |
|  | Mid–HD | 0.005±0.001 |  |  | Post-HD | 0.281 | –0.009 to 0.002 |
|  | Post–HD | 0.009±0.002 |  |  |  | | |
| HbR | Pre–HD | 0.001±0.001 | 0.658 | 0.494 | Mid–HD | 0.553 | –0.001 to 0.003 |
|  |  |  |  |  | Post–HD | 1.000 | –0.003 to 0.003 |
|  | Mid–HD | –0.001±0.000 |  |  | Post-HD | 1.000 | –0.004 to 0.002 |
|  | Post–HD | 0.001±0.001 |  |  |  | | |
| HbT | Pre–HD | 0.005±0.002 | 1.913 | 0.166 | Mid–HD | 1.000 | –0.004 to 0.006 |
|  |  |  |  |  | Post–HD | 0.638 | –0.012 to 0.004 |
|  | Mid–HD | 0.004±0.001 |  |  | Post-HD | 0.258 | –0.012 to 0.002 |
|  | Post–HD | 0.009±0.003 |  |  |  | | |
| HbD | Pre–HD | 0.004±0.003 | 1.012 | 0.370 | Mid–HD | 1.000 | –0.009 to 0.006 |
|  |  |  |  |  | Post–HD | 0.601 | –0.011 to 0.004 |
|  | Mid–HD | 0.006±0.002 |  |  | Post-HD | 0.808 | –0.008 to 0.003 |
|  | Post–HD | 0.008±0.003 |  |  |  | | |

* indicates statistical significance (p<0.05)

11) channel 11

| Measures | Acquisition time | Changes in concentrations (µM) | F | *p-value* | Pairwise comparisons | *p-value* | 95% confidence interval |
| --- | --- | --- | --- | --- | --- | --- | --- |
| HbO_2_ | Pre–HD | 0.001±0.000 | 3.878 | *0.036 | Mid–HD | 1.000 | –0.004 to 0.002 |
|  |  |  |  |  | Post–HD | *0.020 | –0.007 to 0.000 |
|  | Mid–HD | 0.002±0.001 |  |  | Post-HD | 0.368 | –0.007 to 0.002 |
|  | Post–HD | 0.005±0.001 |  |  |  | | |
| HbR | Pre–HD | 0.002±0.001 | 0.030 | 0.931 | Mid–HD | 1.000 | –0.005 to 0.004 |
|  |  |  |  |  | Post–HD | 1.000 | –0.003 to 0.003 |
|  | Mid–HD | 0.002±0.002 |  |  | Post-HD | 1.000 | –0.005 to 0.005 |
|  | Post–HD | 0.002±0.001 |  |  |  | | |
| HbT | Pre–HD | 0.003±0.001 | 2.731 | 0.088 | Mid–HD | 0.742 | –0.005 to 0.002 |
|  |  |  |  |  | Post–HD | 0.080 | –0.008 to 0.000 |
|  | Mid–HD | 0.004±0.001 |  |  | Post-HD | 0.733 | –0.007 to 0.003 |
|  | Post–HD | 0.007±0.002 |  |  |  | | |
| HbD | Pre–HD | 0.000±0.001 | 1.049 | 0.339 | Mid–HD | 1.000 | –0.008 to 0.006 |
|  |  |  |  |  | Post–HD | 0.122 | –0.008 to 0.001 |
|  | Mid–HD | 0.000±0.003 |  |  | Post-HD | 1.000 | –0.011 to 0.005 |
|  | Post–HD | 0.003±0.002 |  |  |  | | |

* indicates statistical significance (p<0.05)

12) channel 12

| Measures | Acquisition time | Changes in concentrations (µM) | F | *p-value* | Pairwise comparisons | *p-value* | 95% confidence interval |
| --- | --- | --- | --- | --- | --- | --- | --- |
| HbO_2_ | Pre–HD | 0.007±0.002 | 1.732 | 0.198 | Mid–HD | 1.000 | –0.005 to 0.007 |
|  |  |  |  |  | Post–HD | 0.676 | –0.026 to 0.009 |
|  | Mid–HD | 0.006±0.001 |  |  | Post-HD | 0.446 | –0.026 to 0.007 |
|  | Post–HD | 0.016±0.007 |  |  |  | | |
| HbR | Pre–HD | 0.001±0.001 | 1.781 | 0.192 | Mid–HD | 1.000 | –0.003 to 0.002 |
|  |  |  |  |  | Post–HD | 0.459 | –0.014 to 0.004 |
|  | Mid–HD | 0.001±0.001 |  |  | Post-HD | 0.684 | –0.014 to 0.005 |
|  | Post–HD | 0.006±0.004 |  |  |  | | |
| HbT | Pre–HD | 0.008±0.002 | 2.121 | 0.153 | Mid–HD | 1.000 | –0.006 to 0.007 |
|  |  |  |  |  | Post–HD | 0.479 | –0.038 to 0.010 |
|  | Mid–HD | 0.008±0.002 |  |  | Post-HD | 0.412 | –0.038 to 0.009 |
|  | Post–HD | 0.022±0.010 |  |  |  | | |
| HbD | Pre–HD | 0.007±0.002 | 0.626 | 0.481 | Mid–HD | 1.000 | –0.005 to 0.008 |
|  |  |  |  |  | Post–HD | 1.000 | –0.017 to 0.011 |
|  | Mid–HD | 0.005±0.001 |  |  | Post-HD | 0.990 | –0.018 to 0.008 |
|  | Post–HD | 0.010±0.005 |  |  |  | | |

* indicates statistical significance (p<0.05)

13) channel 13

| Measures | Acquisition time | Changes in concentrations (µM) | F | *p-value* | Pairwise comparisons | *p-value* | 95% confidence interval |
| --- | --- | --- | --- | --- | --- | --- | --- |
| HbO_2_ | Pre–HD | 0.003±0.001 | 4.694 | *0.032 | Mid–HD | 0.588 | –0.004 to 0.001 |
|  |  |  |  |  | Post–HD | 0.060 | –0.015 to 0.000 |
|  | Mid–HD | 0.004±0.001 |  |  | Post-HD | 0.188 | –0.013 to 0.002 |
|  | Post–HD | 0.010±0.003 |  |  |  | | |
| HbR | Pre–HD | 0.000±0.000 | 0.999 | 0.362 | Mid–HD | 1.000 | –0.003 to 0.003 |
|  |  |  |  |  | Post–HD | 0.439 | –0.001 to 0.003 |
|  | Mid–HD | 0.000±0.001 |  |  | Post-HD | 0.769 | –0.002 to 0.005 |
|  | Post–HD | –0.001±0.001 |  |  |  | | |
| HbT | Pre–HD | 0.003±0.001 | 3.100 | 0.067 | Mid–HD | 1.000 | –0.006 to 0.003 |
|  |  |  |  |  | Post–HD | 0.085 | –0.013 to 0.001 |
|  | Mid–HD | 0.005±0.001 |  |  | Post-HD | 0.439 | –0.012 to 0.003 |
|  | Post–HD | 0.009±0.002 |  |  |  | | |
| HbD | Pre–HD | 0.003±0.001 | 4.840 | *0.029 | Mid–HD | 1.000 | –0.004 to 0.002 |
|  |  |  |  |  | Post–HD | 0.060 | –0.017 to 0.000 |
|  | Mid–HD | 0.004±0.001 |  |  | Post-HD | 0.146 | –0.016 to 0.002 |
|  | Post–HD | 0.011±0.003 |  |  |  | | |

* indicates statistical significance (p<0.05)

.

14) channel 14

| Measures | Acquisition time | Changes in concentrations (µM) | F | *p-value* | Pairwise comparisons | *p-value* | 95% confidence interval |
| --- | --- | --- | --- | --- | --- | --- | --- |
| HbO_2_ | Pre–HD | 0.006±0.002 | 0.896 | 0.352 | Mid–HD | 1.000 | –0.005 to 0.006 |
|  |  |  |  |  | Post–HD | 1.000 | –0.097 to 0.045 |
|  | Mid–HD | 0.005±0.001 |  |  | Post-HD | 1.000 | –0.006 to 0.005 |
|  | Post–HD | 0.032±0.028 |  |  |  | | |
| HbR | Pre–HD | 0.001±0.001 | 0.070 | 0.803 | Mid–HD | 1.000 | –0.004 to 0.005 |
|  |  |  |  |  | Post–HD | 1.000 | –0.031 to 0.025 |
|  | Mid–HD | 0.001±0.001 |  |  | Post-HD | 1.000 | –0.033 to 0.026 |
|  | Post–HD | 0.004±0.011 |  |  |  | | |
| HbT | Pre–HD | 0.007±0.002 | 1.927 | 0.175 | Mid–HD | 1.000 | –0.005 to 0.007 |
|  |  |  |  |  | Post–HD | 0.549 | –0.083 to 0.025 |
|  | Mid–HD | 0.006±0.002 |  |  | Post-HD | 0.499 | –0.083 to 0.024 |
|  | Post–HD | 0.036±0.021 |  |  |  | | |
| HbD | Pre–HD | 0.005±0.003 | 0.402 | 0.533 | Mid–HD | 1.000 | –0.008 to 0.008 |
|  |  |  |  |  | Post–HD | 1.000 | –0.117 to 0.070 |
|  | Mid–HD | 0.005±0.002 |  |  | Post-HD | 1.000 | –0.117 to 0.071 |
|  | Post–HD | 0.028±0.037 |  |  |  | | |

* indicates statistical significance (p<0.05)

15) channel 15

| Measures | Acquisition time | Changes in concentrations (µM) | F | *p-value* | Pairwise comparisons | *p-value* | 95% confidence interval |
| --- | --- | --- | --- | --- | --- | --- | --- |
| HbO_2_ | Pre–HD | 0.014±0.004 | 2.487 | 0.105 | Mid–HD | 1.000 | –0.014 to 0.013 |
|  |  |  |  |  | Post–HD | 0.210 | –0.034 to 0.005 |
|  | Mid–HD | 0.014±0.004 |  |  | Post-HD | 0.353 | –0.036 to 0.008 |
|  | Post–HD | 0.028±0.007 |  |  |  | | |
| HbR | Pre–HD | 0.002±0.002 | 2.084 | 0.134 | Mid–HD | 1.000 | –0.009 to 0.010 |
|  |  |  |  |  | Post–HD | 0.119 | –0.001 to 0.015 |
|  | Mid–HD | 0.002±0.003 |  |  | Post-HD | 0.416 | –0.004 to 0.017 |
|  | Post–HD | –0.005±0.003 |  |  |  | | |
| HbT | Pre–HD | 0.016±0.005 | 0.762 | 0.471 | Mid–HD | 1.000 | –0.019 to 0.019 |
|  |  |  |  |  | Post–HD | 0.492 | –0.021 to 0.006 |
|  | Mid–HD | 0.016±0.006 |  |  | Post-HD | 1.000 | –0.027 to 0.013 |
|  | Post–HD | 0.023±0.005 |  |  |  | | |
| HbD | Pre–HD | 0.012±0.004 | 3.312 | 0.063 | Mid–HD | 1.000 | –0.015 to 0.013 |
|  |  |  |  |  | Post–HD | 0.154 | –0.048 to 0.005 |
|  | Mid–HD | 0.013±0.004 |  |  | Post-HD | 0.228 | –0.049 to 0.008 |
|  | Post–HD | 0.033±0.010 |  |  |  | | |

* indicates statistical significance (p<0.05)
